# Patient and public involvement and engagement in the development of innovative patient-centric early phase dose-finding trial designs

**DOI:** 10.1186/s40900-024-00599-7

**Published:** 2024-06-19

**Authors:** Emily Alger, Mary Van Zyl, Olalekan Lee Aiyegbusi, Dave Chuter, Lizzie Dean, Anna Minchom, Christina Yap

**Affiliations:** 1 Clinical Trial and Statistics Unit, Institute of Cancer Research, London, UK; 2 Drug Development Unit, Royal Marsden/Institute of Cancer Research, Sutton, London, UK; 3 Centre for Patient Reported Outcomes Research, Institute of Applied Health Research, College of Medical and Dental Sciences, University of Birmingham, Birmingham, UK; 4 National Institute for Health and Care Research (NIHR) Birmingham Biomedical Research Centre, University of Birmingham, Birmingham, UK; 5 Advocate Forum, NCRI - National Cancer Research Institute, London, UK

**Keywords:** Patient-Reported Outcomes, Dose-finding, Phase I, Trial design, Patient engagement

## Abstract

**Background:**

In light of the FDA’s Project Optimus initiative, there is fresh interest in leveraging Patient-reported Outcome (PRO) data to enhance the assessment of tolerability for investigational therapies within early phase dose-finding oncology trials. Typically, dose escalation in most trial designs is solely reliant on clinician assessed adverse events. Research has shown a disparity between patients and clinicians when assessing whether an investigational therapy is tolerable, leading to the recommendation of potentially intolerable doses for further investigation in subsequent trials.

It is also increasingly recognized that patient and public involvement and engagement (PPIE) plays a pivotal role in enriching trial design and conduct. However, to our knowledge, no PPIE has explored the optimal integration of PROs in the development of advanced statistical trial designs within early phase dose-finding oncology trials.

**Methods:**

A virtual PPIE session was held with nine participants on 18th October 2023 to discuss the incorporation of PROs within a dose-finding trial design. This cross disciplinary session was developed and led by a team of statisticians, clinical specialists, qualitative experts, and trial methodologists. Following the session, in-depth perspectives were provided by two patient advocates who actively engaged in the PPIE session. We discuss the importance of PPIE in shaping advanced dose-finding trial designs, share insights from patients on integrating PROs to inform treatment tolerability, and present a template for meaningful patient involvement in trial design development.

**Results:**

Participants generally supported the introduction of PROs within dose-finding trials but showed some apprehensiveness as to how PROs may reduce the size of the recommended dose (and potentially efficacious effect). Some participants shared that they may be reluctant to record the real severity of their symptoms via PROs if it would mean that they would have to discontinue treatment. They discussed that PROs could be used to assess tolerability rather than toxicity of a dose.

**Conclusions:**

Amplifying patient voice in the development of patient-centric dose-finding trial designs is now essential. This paper offers an exemplary illustration of how trialists and methodologists can effectively incorporate patient voice in the future development of advanced dose-finding trial designs.

**Supplementary Information:**

The online version contains supplementary material available at 10.1186/s40900-024-00599-7.

## Abstract (plain language summary)

The aim of dose-finding oncology trials is to make sure a treatment is safe, understand its side effects, and recommend the right dose (or doses) for future clinical trials. Traditionally, a patient’s tolerance to treatment is assessed by doctors who evaluate toxicities (side-effects) using established grading guidelines. Research has shown that doctors might not identify all the side effects that patients actually experience during a trial.

There is growing interest in the introduction of patient-reported outcomes (PROs) within dose-finding trials. PROs are reports of a patient’s health and well-being experiences which come directly from the patient themselves, usually assessed using a questionnaire.

In a dose-finding trial, we start with a low dose of a drug and increase it until too many patients have severe side effects. The highest safe dose is then investigated in a later phase trial.   We are suggesting a new way to do these trials. We want to look at both what doctors see as severe side effects and what patients say. This enables us to recommend a dose that balances both perspectives. We would also like to ask patients what level of risk they are comfortable with regarding severe side effects.

In this paper, we highlight the importance of involving patients in creating advanced dose-finding trial designs, particularly with PROs to help decide whether a dose is tolerable for patients. We also share findings of a patient and public involvement and engagement session and provide a guide for meaningful patient involvement in developing trial designs.

**Supplementary Information:**

The online version contains supplementary material available at 10.1186/s40900-024-00599-7.

## Background

Patient and public involvement and engagement (PPIE) ensures that the voice of patients and their advocates inform the conception and development of clinical trial designs [1], contributing to the collection of an improved quality of data and increased patient adherence to the trial. Research has highlighted that, although uncommon, valuable opportunities for patient engagement exist and should be encouraged within the early phase dose-finding oncology trial setting.

### Dose-finding oncology trials

Dose-finding oncology trials (DFOTs) are a crucial step in early clinical development. These trials assess the safety and tolerability of novel anti-cancer therapies across multiple doses. By employing multiple interim analyses, researchers can dynamically test different doses during the trial. Adaptive decision making is based on accumulating preliminary safety and clinical data. This adaptive strategy enhances efficiency and enriches our understanding of an investigational therapy based on emerging patient responses, guiding the selection of optimal doses for potential exploration in subsequent trials. In Phase I cancer trials, various new anticancer therapies, including drugs, radiotherapy, cell therapies and biologics, can be collectively referred to as investigational therapies or novel therapeutic approaches [2]. Henceforth, within this article we will use the term "investigational therapy" to emphasise the investigational nature of these novel therapies in early phase clinical research.

### Patient-reported outcomes

Research has highlighted pitfalls of current practice to tolerability assessment within trials, including clinicians potentially underreporting adverse events compared to a patients’ own assessments of tolerability [3]. Recommendations from new Methodology for the Development of Innovative Cancer Therapies (MDICT) Taskforce guidelines [4], encourage investigators to consider the toxicity burden of new oncology drugs on patients. There is growing interest in integrating Patient-Reported Outcomes (PROs) to enrich our understanding of an investigational therapy’s tolerability profile within early phase trials [4–6].

A PRO is defined as “any report of the status of a patient’s health condition that comes directly from the patient, without interpretation of the patient’s response by a clinician or anyone else” [7]. PROs are readily incorporated within later phase trials, with research showing that the integration of PROs within later phase trials has been associated with improved survival [8]. Within dose-finding trials, the new standardised PRO measure (PROM) PRO-Common Terminology Criteria for Adverse Events (PRO-CTCAE) has been developed by the National Cancer Institute so that patients can self-report symptomatic toxicities via a questionnaire [9]. However, a recent review has suggested that only 5.3% of DFOTs included PROs as an outcome in their trial [5]. Research has shown for those trials that included PROs, they are rarely incorporated to guide dose-escalation decisions in DFOTs (2.9%) [10].

### PPIE for the development of advanced trial designs

The current landscape reveals a notable gap in understanding how to effectively embed PPIE within statistical methodology [11]. There has been a rise in the adoption of advanced model-based and model-assisted trial designs in DFOTs [12]. While these designs enhance efficiency, they come at the cost of increased complexity [13]. Integrating PROs into dose-finding trials requires the creation of smarter yet intricate designs [14–18]. Trial designs could incorporate PROs to dynamically inform dose decisions throughout the trial or influence the final recommended dose(s). Effectively communicating the statistical concepts and workings of these complex designs to a lay audience is crucial for facilitating meaningful PPIE. Exploring patient’s unique insights and lived experience of dose-finding trials is particularly crucial as we consider the patient-centred realm of PROs, where we look to encapsulate elements that are important to dose-finding oncology trial patients.

Particular focus has been placed on the importance of PPIE as we look to incorporate patient-reported outcomes (PROs) within dose-finding trials [19]. Their introduction within the early phase setting is contingent on the support of patients and their advocates. For example, the incorporation of PROs within early phase trials would require patients to record their symptoms, which may increase patient burden [20]. It also requires an alignment in patient and trialist objectives within the Phase I setting.

Within this article we present a case-study for a PPIE session held to discuss the exemplary integration of PROs within the PRO-CRM trial design and utility extension U-PRO-CRM [14, 21]. At the PPIE session, we discussed how to define an intolerable patient toxicity, as identified through PROs, and how we could escalate doses using the rate of patient toxicity in conjunction with clinician assessed toxicities. We expected PPIE discussions to inform future research directions for the field of PRO-integrated trial designs [22, 23], triggering the development of new advanced patient-empowered trial designs.

This paper describes how PPIE was successfully embedded within a statistical methodology project. It provides a template for the organisation of the event, consolidation of research outputs and determination of future directions to support statistical methodologists coordinating their own PPIE activities.

Previous published PPIE within early phase trials has focused on discussing preferences of PRO collection strategies and attitudes towards PRO integration [24]. However, to our knowledge, the session detailed in this paper is the first to discuss a statistical methodology project within the space of early-phase DFOTs.

## Methods

### Patient engagement to inform the development of a new novel trial design

A virtual PPIE session, that lasted an hour and a half, was held with nine participants on 18th October 2023. As well as reflecting on the insights provided by participants at the PPIE session, the aim of the session was to explore how PPIE could be embedded within a statistical methodology project and identify the lessons to be learnt from the session. We anticipated that discussions would inform future research directions in the field of PROs in early phase trials.

Potential participants for the PPIE event were contacted internally via the Institute of Cancer Research’s Drug Development Unit (which runs Phase I trials) and externally, via co-author connections and the National Cancer Research Institute (NCRI) Advocate Forum. Potential participants were eligible to contribute to this PPIE session if they had lived experience within a clinical trial or were experienced patient partners in the Phase I setting. Eleven prospective participants shared an interest in attending the PPIE session and were asked to share their availability via an online form, with the session scheduled for the most popular slot. Nine participants were able to make the scheduled time and two prospective participants were unable to attend due to scheduling conflicts. At first instance, patients enrolled in a Phase I trial at the Institute of Cancer Research’s Drug Development Unit were invited to participate in the session to ensure patients with lived experience in dose-finding trials were engaged with discussions. To ensure we engaged participants with a diverse range of expertise, we also encouraged the participation of other patient stakeholders, including patient advocates.

A PowerPoint presentation and Zoom polls were created for participants to answer pre-set questions. The presentation was developed and refined following a practice presentation with statisticians and clinicians. Questions were curated and reviewed by the team before the session. Strategies were developed to foster an engaging atmosphere, including allotted time for participant introduction, use of Zoom’s “hands up” feature, and appointing an experienced chair with PPIE activity, MVZ (an advanced nurse practitioner) to lead discussions separate to the presenter. Team members EA, MVZ, AM and CY hosted the PPIE session.

**Fig. 1:**
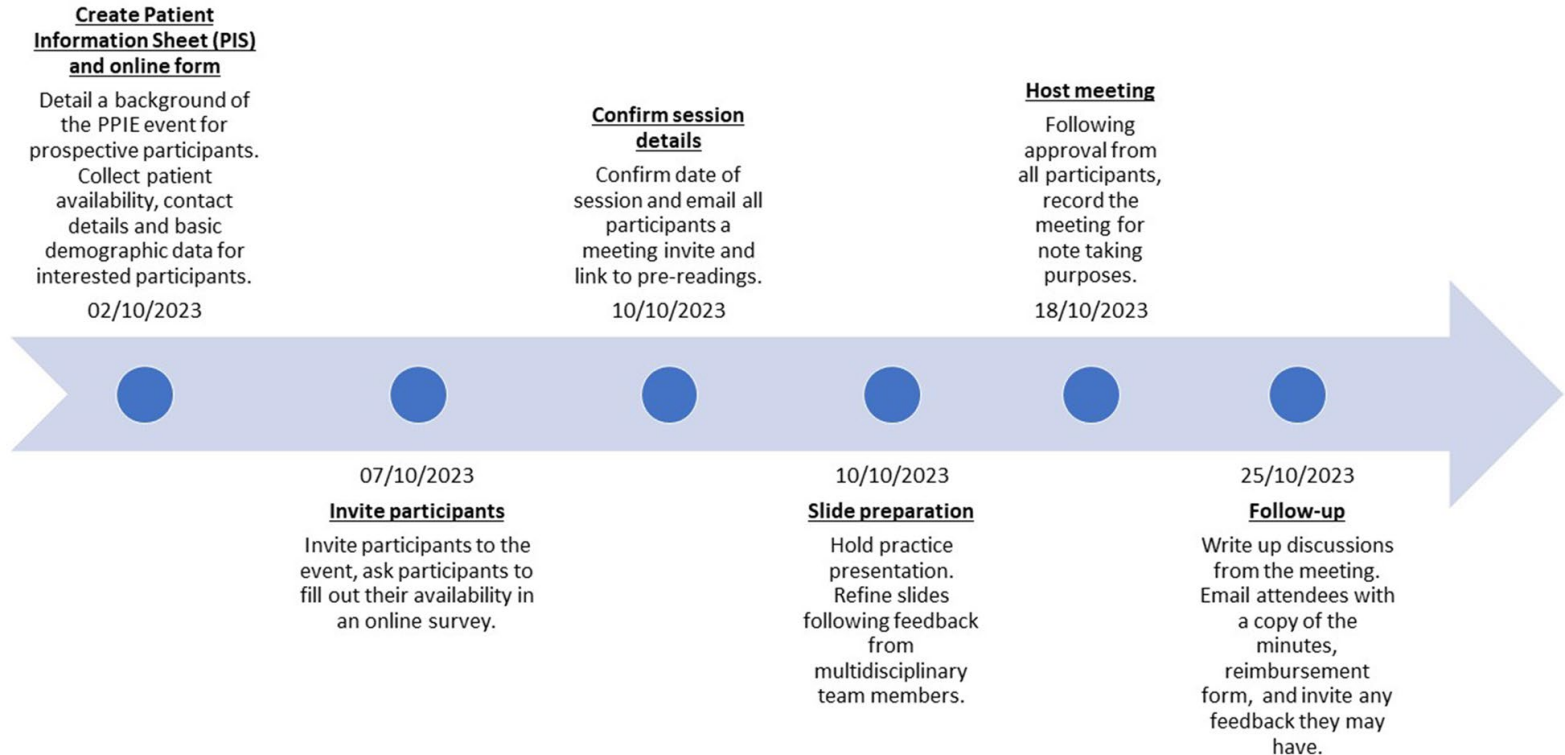
Organisational timeline for PPIE event, from inception to completion

One week before the session, pre-reading materials on dose-finding trials and PROs were distributed to participants for a brief overview of the topic [25, 26]. Following the session, minutes detailing the discussions were sent to participants for approval alongside a reimbursement form. A timeline of the organization for this PPIE event is presented in Fig. 1.

## Results

Participants were based in the UK and Canada. The majority of attendees (66.7%) were at least 65 years old and just over half (55.5%) were female. All participants were white. Six participants (66.7%) had participated in a clinical trial, and four (44.4%) had experience of a Phase I trial. Most (66.6%) were a novice or intermediate patient partner, with introductory or moderate experience as a patient partner in Phase I trials. Two discussants identified themselves as an experienced patient advocate. Characteristics for participants who attended the PPIE session are presented in Table 1.

**Table 1:** Participant characteristics. Novice Patient Partner: Limited or introductory experience as a patient partner in Phase I trials, Intermediate Patient Partner: Some experience as a patient partner in Phase I trials, with a moderate level of involvement, Experienced Patient Partner: Significant experience and active involvement as a patient partner in Phase I trials, Advocate or Leader: Patient partners who have taken on leadership roles, actively advocating for patient interests, and contributing substantially to Phase I trial processes

Demographic	*N* = 9 (%)
**Age (years)**	
25–34	1 (11.1)
35–44	1 (11.1)
55–64	1 (11.1)
65 or older	6 (66.7)
**Sex**	
Female	5 (55.6)
Male	4 (44.4)
**Ethnicity**	
White	9 (100)
**Experience in clinical trials**	
Participant in a clinical trial	6 (66.7)
None	3 (33.3)
**Experience in Phase I clinical trials**	
Participant in a Phase I clinical trial	4 (44.4)
None	5 (55.6)
**Experience as a patient partner in Phase I clinical trials**	
Novice Patient Partner	3 (33.3)
Intermediate Patient Partner	3 (33.3)
Experienced Patient Partner	1 (11.1)
Advocate or Leader	1 (11.1)
Prefer not to say	1 (11.1)

### PPIE insights

At the start of the PPIE session, we defined PROs and current limitations to tolerability assessment. Seven participants (77.8%) agreed or strongly agreed that asking patients to self-identify their symptoms would add useful information to dose-finding clinical trials. Six participants (66.7%) also agreed that PROs should be used together with clinician assessed toxicities to inform dose-escalation decisions.

#### Assessing patient adverse events

Many participants agreed that it was “essential” to listen to a patient’s viewpoint on symptoms, however some had concerns regarding the frequency of PRO collection and the size of the questionnaire. Some participants suggested that PROs were a useful reminder to “help me remember side effects I may have forgotten during the period between doses” and that frequent PRO collection would prevent patients from forgetting the severity of side effects. Whilst some participants thought we “need to record all side effects”, other participants were concerned by the length of the PRO-CTCAE, suggesting that “there’d be an awful lot of things and boxes to tick – an overwhelming number”.

#### Patient tolerability levels

Under the conventional dose-efficacy paradigm, it is typically assumed that as dose increases, so too does its efficacy. Nevertheless, this might not necessarily hold true for modern immunotherapies or targeted agents [27]. For the PRO-CRM and U-PRO-CRM trial designs, PROs are utilised in conjunction with the toxicities assessed by a clinician. Dose-escalation decisions rely upon both the rate of clinician assessed toxicity and rate of patient assessed toxicity. Therefore, for such designs, the incorporation of PROs looks to inform the selection of an admissible set of more tolerable (and potentially lower) doses for investigation in later phase trials.

Participants were mindful about the potential subjectivity of PROs. Whilst some recognised that “[A smaller dose] can be as effective as a higher dose without the side effects”, after discussing the PRO-CRM and U-PRO-CRM design, some participants were concerned that “if individuals are going to report their side effects, and that’s going to influence dose going ahead, what about the fact that everyone reacts differently?”. It was also recognised that “past illnesses, comorbidities will affect how people report”.

#### Impact of PROs on dose decisions and efficacy

Participants discussed whether patients would be fully transparent about the severity of symptoms if the trial offered the last line of investigational therapy. This was a concern for some participants who reflected that “many patients would be reluctant to drop out of the treatment [investigational therapy], unless the clinicians thought that the side effect itself could be life threatening”. Another participant highlighted that “if you were to lower the dose. I feel that it would be a worry that it might not be as effective”. When discussing the possibility of discontinuing an investigational therapy following severe side effects, one discussant suggested that if they were “on a clinical trial and this is my last chance of treatment that might help me, I’m going to tolerate severe pain and probably downplay a little bit [side effects] to the clinicians”.

#### Toxicity vs. tolerability

There was also a suggestion to clarify what is meant by a patient toxicity. It was suggested that instead of using PROs to identify toxic doses, it should be used to identify intolerable doses. It was generally thought that “the clinicians should be the ones to define toxicity, but the patient should be the ones to define how tolerable”. Participants discussed at what point an unpleasant side effect would become “unbearable” – to the point where a patient would refuse further investigational therapy. Instead of defining a single unacceptable level of toxicity using the PRO-CTCAE criteria, one participant suggested that instead patients are asked if “this level [dose] of toxicity would stop them taking part in the trial”.

### Reflections: Learned experiences of running PPIE sessions within the early phase setting

Coordinating this session with a cross-disciplinary team was incredibly beneficial. The allocation of a chair with a clinical background ensured that discussions were accessible and led by an expert with extensive experience of communicating with patients. The active involvement of additional clinicians, statisticians and PRO methodologists was instrumental in overseeing and guiding discussions and questions pertaining to current clinical practices and model-based dose-finding trial designs. It also ensured that the contents and concepts discussed during the session were presented in a manner that was accessible and easily comprehensible to a broad audience. This was evident in the successful engagement of patient partners, marked by numerous discussions throughout the session.

## PPIE: Participants’ perspectives

**Fig. 2:**
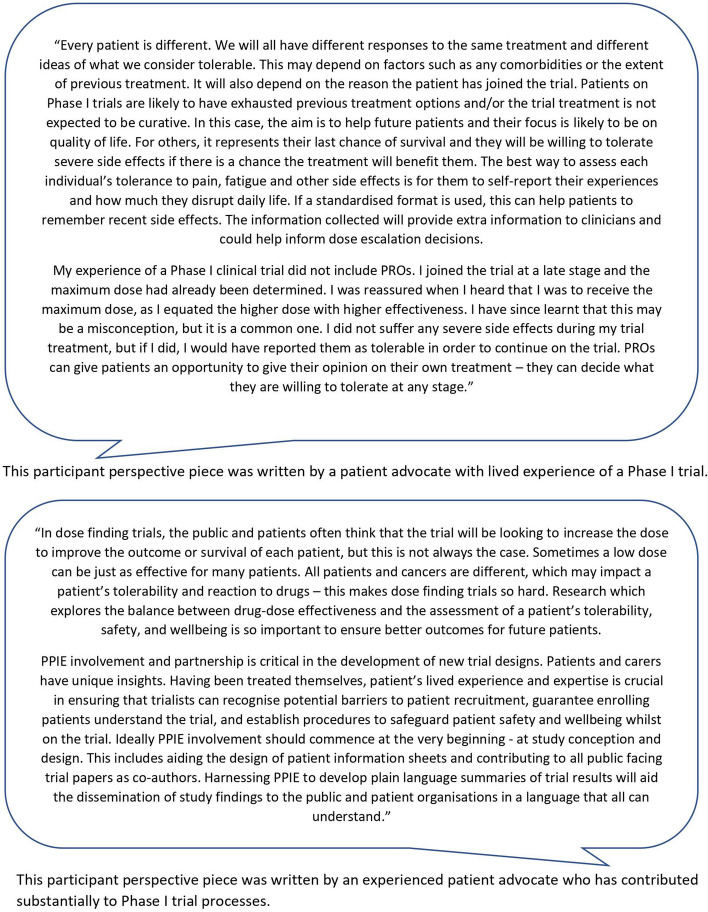
Participant perspective pieces discussing PROs in early phase trials and the importance of PPIE involvement within the development of novel dose-finding trial designs

Figure 2 presents perspective pieces written by two participants who attended the PPIE session.

## Discussion

### PPIE session findings

Participants highlighted concerns that recording their self-assessed side effects accurately may lead to discontinuation of the investigational therapy, potentially reducing their chances of benefiting from the therapy. To promote PRO completion, patients should be informed that individual dosing decisions in Phase I trials are based on protocol-defined adverse events, essential for preventing severe toxicity. Techniques such as intra-patient escalation methods are increasingly advocated to provide additional flexibility to dose escalation trials when implementation is deemed safe [4]. Such escalation routines may enhance the personalisation of dosing for individual patients and treat more patients at the recommended dosage(s). Implementing trial designs which personalise and identify the optimal tolerable dose for each patient by considering the variability in individual tolerability thresholds [28, 29], may encourage patients to diligently complete PROMs. This, in turn, contributes to a more comprehensive understanding of the investigational therapy’s tolerability profile.

Participants also shared their hesitancy about PROs potentially lowering the recommended dose for later phase trials, affecting efficacy. The PPIE session discussion highlights the necessity for PRO-integrated dose-finding trial designs, combining an efficacy endpoint alongside tolerability through both PROs and conventional clinician assessed toxicities. This includes creating seamless Phase I/II designs that dynamically test different doses throughout the trial, based on accumulating tolerability and activity data.

### Future research directions

Following the input of participants at the PPIE session, team members consolidated future study directions to advance the field of trial designs which incorporate PROs.

These include the exploration of PRO-integrated dose-finding trial designs which,

1. Incorporate both efficacy and toxicity assessment.
2. Define intolerable patient toxicities for each patient, allowing for dose-escalation rules to potentially vary among patients.
3. Employ PROs within the dose-optimisation stage of the trial design.
4. Utilise PROs within either interim or final analysis dose recommendations.

Discussions at the meeting indicated that some participants were concerned that PROs could potentially reduce the size of dose recommended for later phase trials. Future research could look to assess how a patient’s tolerability to PRO-determined MTDs would compare to traditional early phase DFOTs designs which rely solely on clinician assessed adverse events.

Future research should continue to explore effective strategies for incorporating patient input into complex statistical methodology projects. This involves the development of novel presentation approaches to ensure information is presented accessibly, including the creation of patient information sheets, slides and scripts for the session [19].

### Strengths and limitations of the session

The virtual nature of this PPIE session encouraged international engagement but did prevent face-to-face discussions, potentially missing out on additional insights that such interactions might have provided.

Providing pre-reading material before the PPIE session helped participants, particularly those with novice or intermediate experience, familiarise themselves with new and advanced concepts on PROs in DFOTs. This pre-reading reflected the richness and depth of the material discussed in the session which included an overview of current practice, PROs and the newly proposed trial design. Participants engaged in some topics more than others, and discussions were often extensive. We would have therefore benefitted from a longer session to cover all areas comprehensively. In hindsight, we recognise it may have been beneficial to conduct this engagement across two separate sessions – first introducing current practice in the early phase setting before expanding on this topic to cover PROs at a later session. Though the time commitment required from participants may be prohibitive.

Our participants encompassed a diverse range of age groups and expertise levels, including individuals with lived experience of Phase I trials. However, whilst we looked to encourage a diverse range of participants to take part in this PPIE session, it is noteworthy that all individuals who expressed interest were white. We hope that future PPIE research which looks to shape trial design will make additional attempts to include a more diverse group of participants. Whilst the FDA provides guidance to help increase engagement of participants from underrepresented groups within later phase trials [30], barriers still remain to engage underserved groups within early phase trials [31].

The majority of participants within this PPIE session were patients within a clinical trial (6/9). Active engagement of patients with lived trial experience is vital to ensure that the perspectives provided are informed by current trial practice and patient viewpoint.

PPIE sessions have a vital role in ensuring that PROs are not just implemented within early phase trials, but to ensure that the implementation is feasible and in line with patient’s own objectives within the trial. These PPIE discussions support contemporary publications which have previously encouraged the tailoring of the PRO-CTCAE into a subset of core symptoms for patient ease [32].

### PPIE in statistical methodology

The incorporation of meaningful PPI can be unclear due to a lack of resources, including successful case studies demonstrating effective implementation [11]. As the strategy for the successful incorporation of PPIE within research continues to be developed [33], case studies such as these can provide recommendations for other researchers looking to introduce PPIE within their research. This project demonstrates the feasibility of PPIE for statistical methodology and the potential of PPIE to originate new research directions within the field of early phase DFOT designs. This research reiterates recommendations of other PPIE in the space of statistical methodology – that engagement is achievable and fruitful if projects are thoroughly considered and organised [34]. Whilst the use of PPIE within statistical projects remains limited, recommendations have been provided by researchers exploring PPIE in the numerical components of trials [35]. Goulao et al. suggest the cultivation of a safe environment, ensuring that participants are listened to and adopting a flexible schedule to allow for additional questions and discussions throughout the session. Involving an experienced PPIE chair within our own session cultivated a safe environment for participants to share their thoughts and confidently raise questions.

## Conclusion

Even before the development of the trial design, engaging patients has the potential to catalyse the direction of future trial designs toward patient-centric considerations – with the potential to inform outcomes, the integration of information in dose decision making, and the frequency of data collection to be considered within the prospective trial design. Continuing to engage patients during the development of trial designs can support methodologists to simplify their complex design and develop lay summaries. Following the adoption of the trial design in practice, such engagement supports the dissemination of the design among potential patients to be enrolled in the study.

Engaging patients in statistical methodology research for PPIE poses unique challenges, especially when compared to applied clinical research. Participants may be eligible for reimbursement for expenses and time. As such any PPIE activity requires financial considerations and budgeting. What’s more, unravelling the advanced statistical concepts of novel dose-finding trials for a lay audience may require the statistical methodologist to exhibit patience, understanding, and strong communication skills. Scheduling the PPIE meeting may be challenging dependent on the size of participants a session looks to engage, however the opportunity to hold sessions virtually does provide additional flexibility. Recruiting participants from ethnically diverse backgrounds remains a challenge in PPIE and clinical trials within the early phase field [31]. Engaging participants, particularly from underserved groups, requires on-going effort to build trust and rapport between prospective participants and the research team.

This article details our experience of optimizing PPIE input. Simplifying complex theories enables us to gather insights from patients on how they envision the utilization of PROs in DFOTs. Influential PPIE is essential as we look to incorporate patient voice into the development of new trial designs. Successful integration not only drives innovation, but also ensures that trials align more closely with what matters to patients, culminating in more patient-centred and impactful research.

### Supplementary Information

Supplementary Material 1. [36].

## Data Availability

No datasets were generated or analysed during the current study.

## Abbreviations

PRO: Patient-reported Outcome
PPIE: Patient and Public Involvement and Engagement
DFOT: Dose-Finding Oncology Trial
PROM: Patient-reported Outcome Measure
PRO-CTCAE: Patient-reported Outcome-Common Terminology Criteria for Adverse Events
MDICT: Methodology for the Development of Innovative Cancer Therapies
NCRI: National Cancer Research Institute

## References

[1] Deverka PA, Bangs R, Kreizenbeck K (2018). A new framework for patient engagement in cancer clinical trials cooperative group studies. JNCI J Natl Cancer Inst.

[2] Ivy SP, Siu LL, Garrett-Mayer E, Rubinstein L (2010). Approaches to phase 1 clinical trial design focused on safety, efficiency, and selected patient populations: a report from the clinical trial design task force of the national cancer institute investigational drug steering committee. Clin Cancer Res.

[3] Seruga B, Templeton AJ, Badillo FEV, Ocana A, Amir E, Tannock IF (2016). Under-reporting of harm in clinical trials. Lancet Oncol.

[4] Araujo D, Greystoke A, Bates S (2023). Oncology phase i trial design and conduct: Time for a change - mdict guidelines 2022. Ann Oncol.

[5] Lai-Kwon J, Yin Z, Minchom A, Yap C (2021). Trends in patient-reported outcome use in early phase dose-finding oncology trials – an analysis of clinicaltrials. Gov Cancer Med.

[6] Basch E, Yap C (2021). Patient-reported outcomes for tolerability assessment in phase I cancer clinical trials. JNCI J Natl Cancer Inst.

[7] United States Department of Health Human Services Food and Drug Administration (2006). Guidance for industry: patient-reported outcome measures: use in medical product development to support labeling claims: draft guidance. Health Qual Life Outc.

[8] Basch E, Deal AM, Dueck AC (2017). Overall survival results of a trial assessing patient-reported outcomes for symptom monitoring during routine cancer treatment. JAMA.

[9] Kluetz PG, Chingos DT, Basch EM, Mitchell SA (2016). Patient-reported outcomes in cancer clinical trials: Measuring symptomatic adverse events with the national cancer institute’s patient-reported outcomes version of the common terminology criteria for adverse events (pro-ctcae). Am Soc Clin Oncol Educ Book.

[10] Alger E, Minchom A, Lee Aiyegbusi O, Schipper M, Yap C (2023). Statistical methods and data visualisation of patient-reported outcomes in early phase dose-finding oncology trials: a methodological review. eClinicalMedicine.

[11] Abell L, Maher F, Begum S (2023). Incorporation of patient and public involvement in statistical methodology research: a survey assessing current practices and attitudes of researchers. Res Involv Engage.

[12] Villacampa G, Patel D, Zheng H (2023). Assessing the reporting quality of early phase dose-finding trial protocols: a methodological review. eClinicalMedicine.

[13] Yap C, Billingham LJ, Cheung YK, Craddock C, O’quigley J (2017). Dose transition pathways: the missing link between complex dose-finding designs and simple decision-making. Clin Cancer Res.

[14] Lee SM, Lu X, Cheng B (2020). Incorporating patient-reported outcomes in dose-finding clinical trials. Stat Med.

[15] Andrillon A, Biard L, Lee SM. Incorporating patient-reported outcomes in dose-finding clinical trials with continuous patient enrollment. J Biopharm Stat. 2023:1–12. 10.1080/10543406.2023.2236216 10.1080/10543406.2023.2236216 PMC10811281 37496233

[16] Wages NA, Nelson B, Kharofa J, Meier T (2022). Application of the patient-reported outcomes continual reassessment method to a phase i study of radiotherapy in endometrial cancer. Int J Biostat.

[17] Wages NA, Lin R. Isotonic phase I cancer clinical trial design utilizing patient-reported outcomes. Stat Biopharm Res. 2024:1–19. 10.1080/19466315.2023.2288013. 10.1080/19466315.2023.2288013 PMC12365655 40842959

[18] Wages NA, Nelson B, Kharofa J, Meier T. Web application for simulating operating characteristics of the pro-crm. 2024. https://uvatrapps.shinyapps.io/pro-crm/. Accessed 31/05/2024

[19] Faulkner SD, Somers F, Boudes M, Nafria B, Robinson P (2023). Using patient perspectives to inform better clinical trial design and conduct: current trends and future directions. Pharma Med.

[20] Kennedy F, Shearsmith L, Ayres M (2021). Online monitoring of patient self-reported adverse events in early phase clinical trials: views from patients, clinicians, and trial staff. Clin Trials.

[21] Yap C, Alger E, Lee S, Cheung YK (2024). 75p u-pro-crm: designing patient-centred dose-finding trials with patient-reported outcomes. ESMO Open.

[22] Doria N, Condran B, Boulos L, Curtis Maillet DG, Dowling L, Levy A (2018). Sharpening the focus: differentiating between focus groups for patient engagement vs. Qualitative research. Res Involv Engage.

[23] Locock L, Boaz A (2019). Drawing straight lines along blurred boundaries: qualitative research, patient and public involvement in medical research, co-production and co-design. Evid Pol.

[24] Lai-Kwon J, Vanderbeek AM, Minchom A (2022). Using patient-reported outcomes in dose-finding oncology trials: surveys of key stakeholders and the national cancer research institute consumer forum. Oncologist.

[25] Le Tourneau C, Lee JJ, Siu LL (2009). Dose escalation methods in phase i cancer clinical trials. JNCI J Natl Cancer Inst.

[26] Bhatnagar V, Dutcus C, Ghiorghiu S, et al. Supporting a patient-centric approach to dose optimization in oncology: The essential role of patient-reported outcomes (pros). 2022. https://friendsofcancerresearch.org/wp-content/uploads/Supporting_Patient-Centric_Approach_Dose_Optimization_Oncology-PROs.pdf. Accessed 3 June 2024.

[27] Patil VM, Noronha V, Joshi A (2019). Low doses in immunotherapy: are they effective?. Cancer Res Stat Treat.

[28] Fernandes LL, Taylor JMG, Murray S (2016). Adaptive phase i clinical trial design using markov models for conditional probability of toxicity. J Biopharm Stat.

[29] Simon R, Rubinstein L, Arbuck SG, Christian MC, Freidlin B, Collins J (1997). Accelerated titration designs for phase i clinical trials in oncology. J Natl Cancer Inst.

[30] Enhancing the diversity of clinical trial populations — eligibility criteria, enrollment practices, and trial designs. Guidance for industry. 2020. https://www.fda.gov/regulatory-information/search-fda-guidance-documents/enhancing-diversity-clinical-trial-populations-eligibility-criteria-enrollment-practices-and-trial. Accessed 03/06/2024.

[31] Chatters R, Dimairo M, Cooper C (2024). Exploring the barriers to, and importance of, participant diversity in early-phase clinical trials: An interview-based qualitative study of professionals and patient and public representatives. BMJ Open.

[32] Janse Van Rensburg HJ, Liu Z, Watson GA (2023). A tailored phase i-specific patient-reported outcome (pro) survey to capture the patient experience of symptomatic adverse events. Bri J Cancer.

[33] Vocal, Medical Research Council. Looking forward: Working with the medical research council towards a public involvement strategy (executive summary). 2023. 9 February 2023. https://www.ukri.org/publications/public-involvement-review/looking-forward-working-with-the-medical-research-council-towards-a-public-involvement-strategy-executive-summary/. Accessed 4th June 2024.

[34] Worboys HM, Broomfield J, Smith A (2023). Incorporation of patient and public involvement in statistical methodology research: Development of an animation. Res Involve Engage.

[35] Goulao B, Bruhn H, Campbell M, Ramsay C, Gillies K (2021). Patient and public involvement in numerical aspects of trials (point): Exploring patient and public partners experiences and identifying stakeholder priorities. Trials.

[36] Staniszewska JS, Brett I, Simera K, Seers C, Mockford S, Goodlad DG, et al. GRIPP2 reporting checklists: tools to improve reporting of patient and public involvement in research. Res Involv Engagem. 2017;3(1). 10.1186/s40900-017-0062-2. 10.1186/s40900-017-0062-2 PMC5611595 29062538

