# Supplementary material for: Patient and public involvement and engagement in the development of innovative patient-centric early phase dose-finding trial designs
Source: Res Involv Engagem. 2024 Jun 19;10:63. doi: 10.1186/s40900-024-00599-7 (PMC11186095; doi:10.1186/s40900-024-00599-7)
Supplement: Supplementary file 1 — Supplementary Material 1. [36]. [file 40900_2024_599_MOESM1_ESM.pdf]

**Section and topic**	**Item**	Reported on Page No
1: Aim	Report the aim of PPI in the study	Page 6
2: Methods	Provide a clear description of the methods used for PPI in the study	Page 6-7
3: Study results	Outcomes—Report the results of PPI in the study, including both positive and negative outcomes	Page 7-11
4: Discussion and conclusions	Outcomes—Comment on the extent to which PPI influenced the study overall. Describe positive and negative effects	Page 12-14
5: Reflections/critical perspective	Comment critically on the study, reflecting on the things that went well and those that did not, so others can learn from this experience	Page 12-14

Table S1 Associated GRIPP2 short form for this manuscript [36].
